# Vitamin E, Alpha-Tocopherol, and Its Effects on Depression and Anxiety: A Systematic Review and Meta-Analysis

**DOI:** 10.3390/nu14030656

**Published:** 2022-02-03

**Authors:** Ainsley Ryan Yan Bin Lee, Areeba Tariq, Grace Lau, Nicholas Wee Kiat Tok, Wilson Wai San Tam, Cyrus Su Hui Ho

**Affiliations:** 1 Yong Loo Lin School of Medicine, National University of Singapore, Singapore 119228, Singapore; e0363343@u.nus.edu (A.R.Y.B.L.); e0602139@u.nus.edu (A.T.); e0421812@u.nus.edu (G.L.); e0474136@u.nus.edu (N.W.K.T.); 2 Alice Lee Centre for Nursing Studies, Yong Loo Lin School of Medicine, National University of Singapore, Singapore 117597, Singapore; nurtwsw@nus.edu.sg; 3 Department of Psychological Medicine, Yong Loo Lin School of Medicine, National University of Singapore, Singapore 119228, Singapore

**Keywords:** antioxidants, micronutrients, vitamin E, major depressive disorder, depression, generalised anxiety disorder, anxiety, health supplementation

## Abstract

Background: Recently, it has been discovered that anti-inflammatory and anti-oxidative pathways play a role in depression and anxiety. Lower serum levels of antioxidants, such as vitamin E, have been implicated in both depression and anxiety. Methods: This PROSPERO-registered systematic review (Reference: CRD42021260058) is reported according to PRISMA guidelines. PubMed, EMBASE, CENTRAL, PsycINFO, and CINAHL were searched from inception to June 2021. Results: Twelve studies were included in this systematic review, and nine in meta-analysis of vitamin E versus placebo. For depression, meta-analysis of 354 participants showed a standardised mean difference of –0.88 (95% CI: –1.54, –0.21; I2 = 87%) favouring vitamin E. For anxiety, meta-analysis of 306 participants showed a standardised mean difference of –0.86 (95% CI: –2.11, 0.40; I2 = 95%) favouring vitamin E. Three of the studies involved a pure comparison of vitamin E against placebo, while others included constituents such as omega-3 fatty acids. Nine of the studies were at low risk of bias, two had some concerns, and one was at high risk of bias. Conclusion: Vitamin E supplementation has shown inconclusive results in ameliorating both depression and anxiety. Containing a reassuring safety profile and low cost, future studies would be of promise, and they would benefit from both larger sample sizes and from excluding other constituents, such as omega-3 fatty acids, from experimental and comparator arms.

## 1. Introduction

There has been a rise in mental health conditions in the last decade. Depressive and anxiety disorders displayed a high prevalence of 3627 and 3715 per 100,000 people, respectively, in 2016, and these disorders also contribute to over 10% of all years lived with disability [1,2]. Depression and anxiety make up a significant portion of the growing global psychiatric burden, and they also have negative implications for morbidity, socio-economic contribution, and quality of life (QOL). Depression is a psychological problem that is characterised by anhedonia, fatigue, low mood, senses of worthlessness and, in more severe cases, suicidal ideations and self-harm [3,4]. Anxiety disorders refer to a spectrum of disorders, from generalised anxiety disorder to phobia-related disorders, thought to be mediated by the sympathetic nervous system and norepinephrine, serotonin, dopamine, and gamma-aminobutyric acid in the central nervous system [5,6].

It is well known that these psychiatric disorders have extremely debilitating effects on individuals. Firstly, these disorders can cause an increase in mortality and reduced life expectancy. It has been observed that 3.5% of all deaths can be attributed to depression and anxiety [7,8]. Secondly, these disorders can lead to a drop in one’s QOL, as the symptoms of these conditions can significantly hamper one’s ability to work and participate in social activities [9]. Finally, these disorders have an all-around deleterious effect on economies [10].

Currently, the mainstays pertaining to treatment of depression and anxiety include medication and psychotherapy [11]. The repertoire of medications includes tricyclic antidepressants (TCAs), selective serotonin reuptake inhibitors (SSRIs), selective serotonin noradrenaline reuptake inhibitors (SNRIs), and other less commonly prescribed medications [12,13]. However, many of these treatments are associated with stigma as well as fear of side effects. This highlights the benefits of examining dietary supplements as a treatment modality for depression, as they are often observed as more natural, and they carry less stigmatisation.

It has been recently shown that anti-inflammatory and anti-oxidative pathways play a role in depression and anxiety. Greater levels of pro-inflammatory cytokines, such as TNFα and IL-6, have been found in patients suffering from depression, and this is potentially due to structural and functional changes being caused in the central nervous system, particularly the hippocampus. Both depression and anxiety disorders have a basis in pro-inflammatory states; these findings have been established in both human and animal models [14,15,16]. As such, in recent years, vitamins and constituents of anti-inflammatory pathways, such as IL-1β, IL-6, and IL-18, have been identified as promising pathways to reduce anxiety and depression. Studies of antioxidant pathways have also postulated that, when stress causes biochemical changes, antioxidants are able to neutralise free radicals and repress the oxidative stress pathway, removing the reactive oxygen species (ROS) and reactive nitrogen species (RNS) that may cause harm to neurons in the brain. Consequently, this process may lead to a reduction in the symptoms of anxiety and depression.

Other studies have demonstrated the possible antidepressant-like effect of vitamin E [17,18,19]. Vitamin E is a nonenzymatic antioxidant that plays a secondary role, alongside enzymatic antioxidants, such as glutathione peroxidase and superoxide dismutase, in reducing oxidant changes resulting from stress. Lower serum levels of antioxidants, such as vitamin E, have been implicated in both depression and anxiety [20]. Common natural sources of vitamin E include nuts and vegetable oils [21]. Several studies have shown that antioxidant supplement therapy is effective in patients with anxiety and depression, as it enhances antioxidant defence in the biological system [22,23]. In addition, vitamin E supplementation has few associated adverse events [24].

To date, there have been no systematic reviews observing the benefits of vitamin E supplementation in depression and anxiety. Thus, this review aims to synthesise the current evidence (about the use of vitamin E in preventing and alleviating symptoms of depression and anxiety) and explore the potential of vitamin E (alpha-tocopherol) as co-adjuvant therapy in the treatment of depression and anxiety disorders.

## 2. Methodology

This systematic review is reported according to the Preferred Reporting Items of Systematic Reviews and Meta-Analyses (PRISMA) guidelines [25]. The following electronic databases were searched from inception to June 2021: Medline, Embase, CINAHL, CENTRAL, and PsycINFO. The citations were downloaded, and duplicates were removed with the EndNote X9 software. The search strategy is detailed in Table 1. This protocol was registered prospectively on PROSPERO, CRD42021260058.

## 3. Study Selection

Two reviewers independently screened the citations manually using EndNote X9, and those that did not fulfil the inclusion criteria were excluded, after which a full-text review was conducted. Those that met the criteria were included in this article, with all differences being solved by consensus.

Studies were included if they (1) involved vitamin E containing regimens as part of any arm, (2) involved participants formally diagnosed with depression and/or anxiety, or with conditions that put them at risk, (3) were controlled studies, and (4) involved the collection of quantitative results related to depression and/or anxiety.

The full inclusion and exclusion criteria are detailed in Table 2. Only original, peer-reviewed, papers were considered. Two reviewers independently conducted a full-text review, and the inclusion discrepancies were discussed and resolved through consensus.

## 4. Data Extraction

Four reviewers independently read the selected articles, and they recorded and extracted data using a structured proforma on Microsoft Excel. Additional quality control of all extracted data was conducted at the end of the data extraction by a statistician who compared the extracted results with published data.

## 5. Quality Assessment

Quality appraisal of all included studies was performed by two reviewers using the Cochrane Risk of Bias 2.0 tool that assesses five domains: bias arising from (1) the randomisation process, (2) deviations from intended interventions, (3) missing outcome data, (4) measurement of the outcome, and (5) bias in the selection of the reported result for randomised controlled studies [26].

## 6. Data Analysis

The extracted data were quantitatively pooled and analysed in Review Manager (RevMan) Version 5.4 [27], following guidelines detailed in the Cochrane Handbook [28]. In studies without standard deviations (SDs), confidence intervals (CIs) were converted to SDs. In studies without relevant baseline data, the simple analysis of the final values method was used. In studies reporting the outcome in different scales, simple unit conversion was conducted. Inverse variance was used to derive the pooled outcomes, and the random-effects model was used to account for between-study variance. Between-study heterogeneity was represented by I^2^ and τ^2^ statistics. I^2^ of <30% was considered to indicate low heterogeneity between studies, while 30% to 60% indicated moderate heterogeneity, and >60% indicated substantial heterogeneity. Two-sided *P* values of <0.05 were considered to indicate nominal statistical significance.

## 7. Results

**Figure 1:**
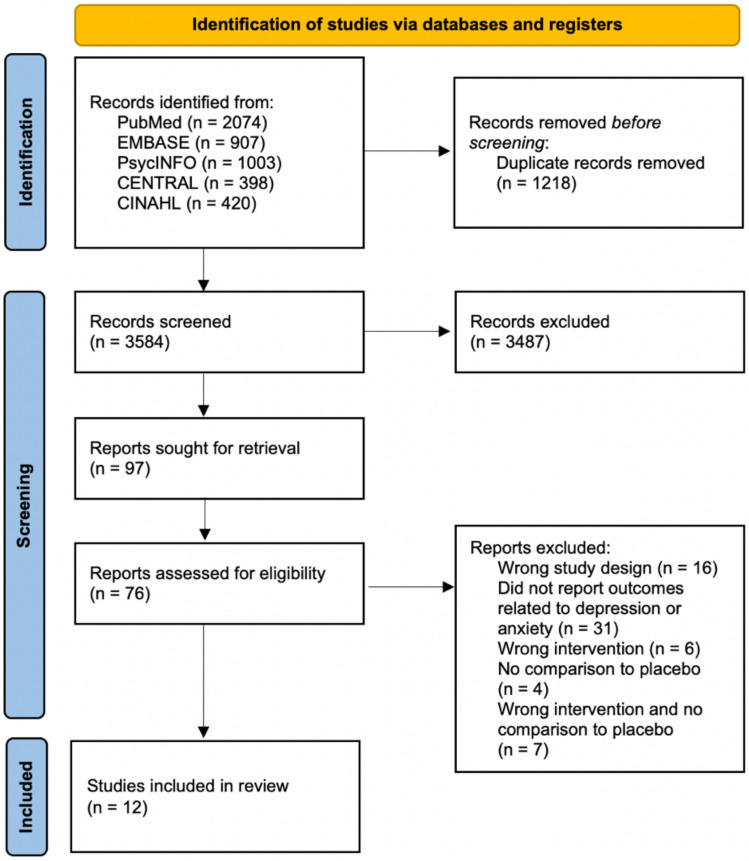
PRISMA flowchart. From 3,584 records, 12 studies were included in this review. [29,30,31,32,33,34,35,36,37,38,39,40].

From 3,584 records, 12 research papers were included in this review. All of the studies were randomised controlled trials. This process is detailed in the PRISMA flowchart in Figure 1.

Four of the studies only included female participants, as they were studying conditions such as post-menopausal depression and Polycystic ovary syndrome (PCOS) [29,30,31,32]. Two studies focused on the elderly population, including both male and female participants [33,34]. As intervention duration varied, data closest to the three-month timepoint were extracted and used in meta-analysis. Six studies reported data between four to 12 weeks [29,30,31,32,35,36], and three reported data at six months [34,37,38]. Detailed characteristics of the included studies are presented in Table 3.

## 8. Drug Constituents

Three of the studies [35,36,39] used only vitamin E as part of the intervention, while the remaining studies used other constituents in addition to vitamin E, such as omega-3 fatty acids, omega-6 fatty acids, and pravastatin [34,36]. Three of the studies [29,31,40] used omega-3 fatty acids among the other constituents. These studies used different forms of vitamin E in their intervention, such as alpha-tocopherol and d-vitamin E, and one study used a silybin-vitamin E-phospholipids complex.

However, none of the studies used vitamin E in combination with any other vitamin as part of the same arm. One study [36] used vitamin C as a comparator, but in a different arm from vitamin E; thus, this study was included.

According to the National Institutes of Health, the recommended daily intake of vitamin E for adults is 15 mg. In comparison, most studies used at least twice the recommended daily intake of vitamin E, with some using 10 to 20 times the recommended amount. Five of the twelve studies used a dose of 268 mg (400 IU) of vitamin E, with one study [33] choosing their dosage based partly on the encouraging results obtained by their colleagues who were treating Juvenile neuronal ceroid lipofuscinoses (JNCL). The same study also noted an improved sense of wellbeing in elderly patients who had received antioxidant therapy, justifying their use of a high dose of vitamin E.

However, it is worth noting that too high a dose of vitamin E might increase the risk of bleeding and hemorrhagic stroke. Therefore, the National Institutes of Health have set an upper limit of 1000 mg/day for adults, pertaining to supplements of either natural or synthetic vitamin E. Yet, one study [38] used a dosage of 2000 IU (1340 mg) of vitamin E, and this amount is close to 90 times the daily recommended intake.

The following section summarises the effects of the respective interventions on measures of depression and/or anxiety. Each outcome is either directly measured using scales specific to depression and/or anxiety, or it is derived from scales used for generic psychiatric assessments. Due to heterogeneity in the reporting of outcomes that were not amenable to pooling, such as the absence of index data, two studies were not included in this meta-analysis; they were analysed qualitatively.

Depression was studied more commonly, with all but one study measuring the effect of vitamin E on depression. Overall, most studies reported an improvement in depressive symptoms post-intervention. Four of these studies used The Beck Depression Inventory (BDI) as a measure of depression. For three of the studies [31,37,38] that used BDI, there was a significant reduction in the score, reflecting an improvement in depression levels for these studies. Another study [40] used BDI as well; however, the study showed no significant changes in depression scores.

One study [29] used the Hamilton Depression Rating Scale, and this study reported significant improvement across each of the measures of depression.

Three of the papers studying depression included solely female participants, as they studied conditions such as PCOS and menopause. One such study [30] used the Greene Climacteric Scale (GCS); this scale is commonly used to measure the symptoms of menopause, which include depression. This study reported a reduction in the severity of depression in the intervention group.

### 8.1. Anxiety

Seven of the studies measured anxiety, with most of these reporting a reduction in anxiety symptoms in the intervention group. Four of the studies [30,33,35,37] showed a statistically significant decrease pertaining to anxiety levels in the intervention group, while the remaining three studies reported no significant improvement in anxiety levels.

Nearly all of the studies used different scales, with the exception of two studies [35,39]; these two used The Brief Psychiatric Rating Scale (BPRS). Both of these studies measured the effect of vitamin E on patients with tardive dyskinesia, which is a side-effect of antipsychotic medications. One of these studies [35] contained a significant reduction in anxiety scores. The other study [39] reported the total score, but not the anxiety subscale results of the BPRS. As the BPRS measures multiple domains, including anxiety and depression, this study could not be included for meta-analysis. However, this study [39] presented no significant effects of vitamin E on the total BPRS score.

### 8.2. Measures of Effect

As mentioned above, there was heterogeneity in the questionnaires and scales used for measuring depression and anxiety used in each study. There were some studies that used scales that were not specifically for measuring depression and anxiety disorders, such as the BPRS. However, since the BPRS is used to measure psychiatric symptoms which include depressed mood and anxiety, we were able to associate a statistically significant improvement in this scale with an improvement in depression and anxiety levels. Thus, we included these studies in our meta-analysis.

Some studies also used multiple scales to measure each outcome, and, as a result, we needed to decide on one scale to represent each outcome. For instance, Jamilian et al. [31] used both the BDI and the General Health Questionnaire-28 (GHQ-28) to measure depression. We chose to use the BDI scores in this case, as BDI is a scale more commonly used to measure depression levels, and it was used in four other studies we included. Furthermore, BDI has a higher level of specificity than GHQ-28, as BDI solely measures depression while GHQ-28 has four sub-components: somatic symptoms, anxiety and insomnia, social dysfunction, and depression.

### 8.3. Synthesis of Results

#### 8.3.1. Depression

**Figure 2:**
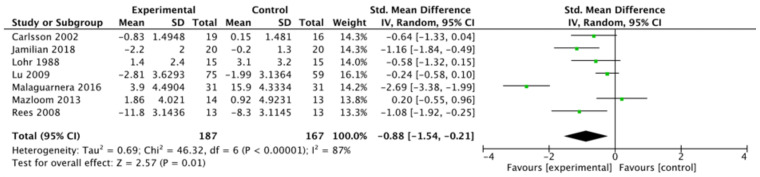
Meta-analysis of studies measuring depression. [29,31,34,35,36,37,38].

With respect to depression, a total of 354 participants were included in the analyses, with 187 in the intervention group, and 167 in the comparator group (Figure 2). Overall, a standardised mean difference of –0.88 (95% CI: –1.54, –0.21; I^2^ = 87%) was noted in the intervention versus the comparator group. Among the studies included in meta-analysis, only Mazloom et al. [36] demonstrated results favouring the control; this was not statistically significant. Three studies demonstrated a significant improvement favouring the experimental group [29,31,37].

As Tolonen et al. [33] and Radzinskii et al. [30] did not report quantitative values of depression, they were not included in the meta-analysis. In Tolonen et al.’s study, significant improvements were noted in both depression (*p* < 0.01) and anxiety (*p* < 0.01) among patients receiving a treatment regimen containing d-alpha-tocopherol, as compared to the control group. Radzinskii et al. reported the proportion of participants experiencing sadness or depression as a component of the Greene Climacteric Scale without quantifiable data. Similarly, favourable results were shown in the group receiving vitamin E compared to placebo. Before the intervention, 51 (82.3%) and 42 (66.7%) of the participants in the vitamin E and placebo groups, respectively, reported sadness or depression, improving to 17 (27.4%) in the vitamin E group, while worsening to 51 (81.0%) in the placebo group.

#### 8.3.2. Anxiety

**Figure 3:**
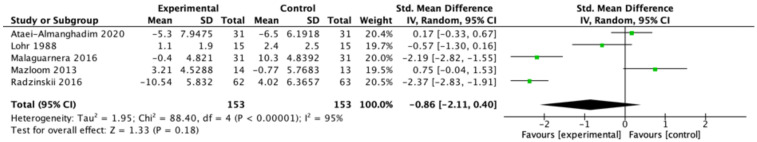
Meta-analysis of studies measuring anxiety. [30,32,35,36,37].

With respect to anxiety, a total of 306 participants were included in the meta-analysis (Figure 3), with 153 in the intervention group, and 153 in comparators. Overall, a standardised mean difference of –0.86 (95% CI: –2.11, 0.40) was noted in the intervention compared to the control group. Three studies [30,35,37] reported improvements in outcomes, with Malaguarnera et al. [37] and Radzinskii et al. [30] being statistically significant. Two studies [32,36] reported improved outcomes in the control group, but these were not statistically significant.

#### 8.3.3. Risk of Bias

**Figure 4:**
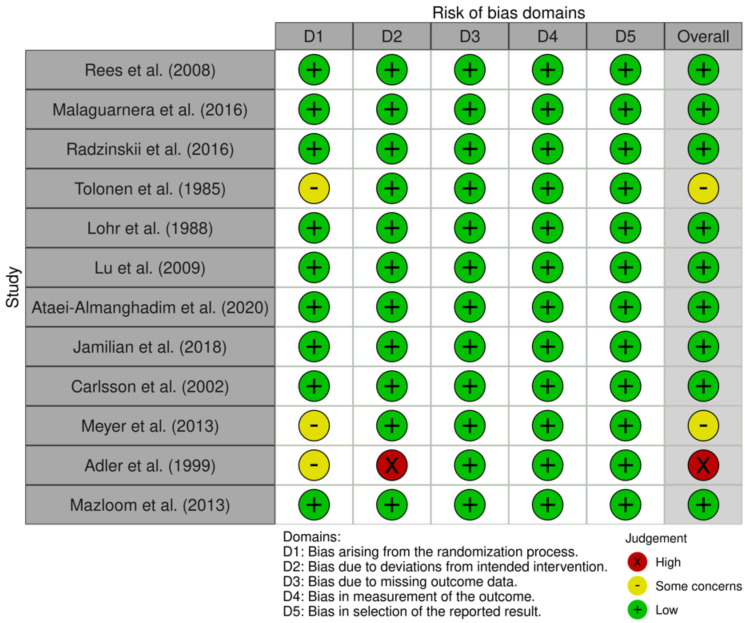
Risk of bias assessment using the Cochrane Risk of Bias 2.0 tool. [29,30,31,32,33,34,35,36,37,38,39,40].

The overall risk of bias (ROB) was judged as ‘low’, as the majority of studies contained a low ROB assessment. Studies done by Tolonen et al. [33] and Carlsson et al. [34] were regarded as having ‘some concerns’, and this was due to a lack of detail regarding the randomisation process. The study reported by Meyer et al. [40] was regarded as having a ‘high’ ROB, as there was a significant percentage of study participants (28%) deemed to violate the trial protocol, and it was not detailed whether an appropriate analysis was used to estimate the effect of adhering to the intervention. The ROB of RCTs is represented in Figure 4

## 9. Discussion

This meta-analysis is the first to focus solely on the effects of vitamin E pertaining to depression and anxiety. Due to the many conflicting studies which have been conducted prior to this analysis, there is a need to consolidate the findings in order to provide guidance on the use of vitamin E. This meta-analysis of randomised controlled trials has shown that, at present, there is some evidence pointing towards a positive effect of vitamin E supplementation on mood outcomes in adults who are at risk of, or clinically diagnosed with, depression. However, results for anxiety have not been statistically significant.

In particular, the combination of vitamin E and omega-3 fatty acids was used as the intervention in three studies [29,31,40]. One study [31] out of these three reported a statistically significant reduction in depression and anxiety symptoms, suggesting that vitamin E and omega-3 fatty acids may have synergistic effects due to postulated roles in the antioxidant and inflammatory pathways [41]. However, the remaining two papers propose that further studies are required to more accurately establish whether the combination of vitamin E and omega-3 fatty acids is effective for reducing symptoms of depression and anxiety.

It is also worth noting that four studies [29,30,31,32], containing interventions on depression and anxiety disorders that were secondary to gynaecological conditions, investigated the effects of vitamin E. Out of these four studies, two [29,31] found a statistically significant reduction in depression and anxiety symptoms, while the other two did not find a statistically significant reduction.

## 10. Limitations of Review

There are some notable challenges to mention with respect to the review of these studies. The majority of the studies used vitamin E in combination with other constituents (e.g., omega-3 fatty acids, silybin, phospholipids), and there was heterogeneity in the interventions and controls (e.g., not using pure vitamin E), which may potentially affect the outcomes noted in the studies. Most of the studies also used vitamin E in doses that were higher than the recommended daily intake of 15 mg for adults, though none of the studies mentioned any adverse effects reported by subjects due to the high doses.

Additionally, there are varying demographics included across the studies. Two of the studies included participants with a mean or median age above 70, while most of the studies varied between 30 to 50 years old. Further, three of the studies included only female participants. The disease conditions studied also differed, such as Malaguernera et al. [37], which studied patients with hepatitis C, and Rees et al. [29], which involved women in the perinatal period. The underlying conditions patients suffer from may intrinsically confound how their depressive and anxiety symptoms respond. In addition, there is a lack of homogeneity in the questionnaires and scales used to assess the outcomes. This lack of homogeneity could be attributed to the different demographics included across the studies. All of the studies were concerned with different overall outcomes (e.g., improvement of menopausal symptoms, improvement of tardive dyskinesia), and there is currently no gold standard pertaining to the measurement of depression and anxiety disorders across all these different demographics.

The small sample sizes in the randomised controlled trials also limited statistical significance, with six out of twelve studies containing less than 50 participants in total. However, no study suffered from a significant attrition rate, or from missing outcome data.

There were trial-level characteristics that varied across studies. Three of the studies involved follow-up durations of more than one year, while nine studies involved intervention and follow-up durations of one year or less. This variance may have contributed to heterogeneity among studies, as longer interventions may result in a greater propensity for either non-compliance over the trial or external factors contributing to amelioration or deterioration in depression and anxiety.

Overall, high heterogeneity was noted among the studies. However, the current landscape does not allow for homogeneity given the lack of consistent methodology across studies. Lastly, due to methodological constraints, we restricted our eligibility criteria to include only those studies published in English or with an English translation available. Therefore, one should take these limitations into consideration while interpreting the results.

Future studies should explore the efficacy of vitamin E, at the recommended dose, in creating antidepressant and anxiolytic effects, in more detail, and these studies should be mindful to carefully detail the vitamin E status of participants at baseline as well as the post-intervention states in order to investigate the relationship between a change in symptoms (of anxiety and depression) and a change in micronutrient status. The current literature suggests that there are potential benefits of having vitamin supplementation, such as improving psychological distress [42]; however, more randomised controlled trials, which include both a larger number of individuals and the use of vitamin E without other constituents, are currently required to further support the recommendation of vitamin E as a therapeutic strategy for managing depression and anxiety disorders. Studies with animal models have also proven the antidepressant effects of vitamin E [17].

## 11. Conclusions

In conclusion, while there is a paucity of high-powered randomised controlled studies evaluating the efficacy of vitamin E supplementation, it has shown promise in ameliorating depression; however, it has demonstrated inconclusive findings for anxiety. Given the stigma associated with established medications and psychotherapy, treatment options consisting of complementary treatments and health supplements may have a pertinent role to play. Existing as a supplement with an assuring safety profile and low cost in most nations, the addition of vitamin E to the armamentarium of therapeutics for depression and anxiety may be beneficial.

## Figures and Tables

**Table 1:** Search terms.

Search Engine	Search Term	No. of Results
EMBASE	(‘depress*’:ti,ab OR ‘anxie*’:ti,ab OR ‘low mood’:ti,ab OR ‘mood disorders’:ti,ab OR ‘psych*’:ti,ab) AND (‘vitamin e’ OR ‘alpha tocopherol’ OR ‘d alpha tocopherol’ OR ‘α tocopherol’)	2074
PubMed	(“depress*”[title/abstract] OR “anxie*”[title/abstract] OR “low mood”[title/abstract] OR “mood disorder”[title/abstract] OR “psych*”[title/abstract]) AND (vitamin e OR alpha-tocopherol OR alpha-tocopherol OR d-alpha-tocopherol OR α-tocopherol)	907
PsycINFO	((depress or depression or depressive or anxiety or low mood or mood disorder or psychiatric or psychiatry or psychology or psychological).ab. or (depress or depression or depressive or anxiety or low mood or mood disorder or psychiatric or psychiatry or psychology or psychological).ti.) and (vitamin e or alpha-tocopherol or alpha-tocopherol or d-alpha-tocopherol).af.	1003
Cochrane (CENTRAL)	(“depress*” OR “anxie*” OR “low mood” OR “mood disorder” OR “psych*”) in Title Abstract Keyword AND (vitamin e OR alpha-tocopherol OR alpha-tocopherol OR d-alpha-tocopherol OR α-tocopherol) in All Text	398
CINAHL	(TI (“depress*” OR “anxie*” OR “low mood” OR “mood disorder” OR “psych*”) OR AB (“depress*” OR “anxie*” OR “low mood” OR “mood disorder” OR “psych*”)) AND TX (vitamin e OR alpha-tocopherol OR alpha-tocopherol OR d-alpha-tocopherol OR α-tocopherol)	420

**Table 2:** Inclusion and exclusion criteria.

	Inclusion	Exclusion
Population	• At risk of or clinically diagnosed with depression AND/OR anxiety disorders	• No patients at risk of or clinically diagnosed with depression AND/OR anxiety disorders
Intervention	• Study involves the use of a vitamin E containing drug as part of any arm, intervention or otherwise, of the study	• Study does not involve the use of a vitamin E containing drug as part of any arm, intervention or otherwise, of the study
Comparator	• Any arm of study that administers a placebo or drug in place of vitamin E	
Outcomes	• Any validated quantitative assessment of severity of anxiety and/or depression • If study only reports qualitative improvement, the results will be assessed with systematic review without meta-analysis	• Study does not have outcomes assessing anxiety and/or depression
Study design	• Randomised controlled trials of any phase • Nonrandomised controlled prospective clinical trials • Long-term follow-up studies • Prospective observational studies	• Systematic reviews and meta-analyses • Non-systematic reviews including literature and scoping reviews • Preclinical studies • Prognostic studies • Retrospective studies • Case reports or series • Editorials, commentaries and letters • Consensus reports
Language	• Articles in English or translated to English	• Articles not in English and with no English translation available

**Table 3:** Characteristics of included studies.

Source	Study design	Participants	Population	Intervention	Comparison (If Any)	Measures of Effect	Duration of Follow-Up	Findings *
Rees et al. (2008) [29]	RCT	26	*Tx:* 31.2 ± 4.4 years old *Pb:* 34.5 ± 3.8 years old Women in the antenatal and postnatal period	6 g containing 27.3% DHA, 6.9% EPA, 3.3% omega-6 fatty acids, 80 mg vitamin E Once per day in divided doses for 6 weeks	Sunola Oil	Edinburgh Postnatal Depression Scale, HDRS, MADRS	6 weeks Data at 6 weeks used for meta-analysis.	Significant improvement in depression with Vitamin E HDRS improved from 19.7 ± 4.8 to 7.9 ± 5.1 in the intervention group versus improvement from 9.0 ± 3.5 to 0.7 ± 5.1 in the placebo group (SMD: –1.08, 95%CI: –1.92, –0.25)†
Radzinskii et al. (2016) [30]	RCT	125	*Tx:* 52.4 ± 5.02 *Pb:* 51.97 ± 4.25 42–60-year-old women with vasomotor and psycho-somatic menopausal symptoms	2 pills (200 mg each) of Amberen daily Amberen contains tocopheryl acetate (vitamin E), ammonium succinate, calcium disuccinate, monosodium l-glutamate, glycine, magnesium disuccinate, zinc difumarate and	Placebo (High purity corn starch)	Greene climacteric test and Spielberger–Hanin test	Data collection every 30 days, followed up for 90 days Data at 3 months used for meta-analysis.	Amberen showed a statistically significant improvement in anxiety, stress resistance and adaptability Spielberger-Hanin test for situational anxiety showed improvement in the Amberen group from 0.52 ± 9.72 to –10.02 ± 7.78 at 90 days versus deprovement from –4.16 ± 10.08 to –0.14 ± 10.05 in the placebo group (SMD: –2.37, 95%CI: –2.83, –1.91)‡
Jamilian et al. (2018) [31]	RCT	40	*Tx:* 22.3 ± 4.7 *Px:* 24.4 ± 4.7	1000 mg omega-3 fatty acids, 400 IU Vit E per day for 12 weeks	Placebo	BDI, general health questionnaire scores, DASS	12 weeks Data at 12 weeks used for meta-analysis.	Co-administration of omega-3 and vitamin E had favourable effects on parameters of mental health After 12 weeks, greater reduction in BDI was noted with Vitamin E –2.2 ± 2.0 versus –0.2 ± 1.3 with placebo (SMD: –1.16, 95%CI: –1.84, –0.49)†
Ataei-Almanghadim et al. (2020) [32]	RCT	93	51.6 ± 5.4 Women with normal menopause	500 mg oral capsule of curcumin Twice a day for 8 weeks	Oral tablets of vitamin E (200 IU/day) Placebo	Hot flashes and anxiety (primary objectives), sexual function, menopausal symptoms and adverse effects (secondary objectives)	4 weeks and 8 weeks after the intervention Data at 8 weeks used for meta-analysis.	Vitamin E had no significant effect on anxiety, sexual function and menopausal symptoms versus placebo After 8 weeks, state anxiety improved from 44.4 ± 13.2 to 39.1 ± 9.9 in the Vitamin E, and 44.9 ± 10.2 to 38.4 ± 9.1 in the placebo group (SMD: 0.17, 95%CI: –0.33, 0.67)‡
Tolonen et al. (1985) [33]	RCT	30	*Tx:* 76.8 (58–90) years old; 26.7% male *Pb:* 76.2 (50–92) years old; 20.0% male Geriatric patients Medications that participants were on were not specified	8 mg of sodium selenate, one 45 μg capsule of ‘Vita-hiven’ (Se yeast in birch ash) and 400 mg of d-alpha-tocopherol (Ido-E) Twice a day for 1 year	Placebo	Sandoz Clinical Assessment Geriatric-scale	Data collection every 2 months Intervention over 1 year	Statistically significant improvements observed in the therapy group compared with the placebo group in both depression (*p* < 0.001) and anxiety (*p* < 0.01) Quantitative results were not available for meta-analysis
Carlsson et al. (2002) [34]	RCT	41	*Tx:* 76.2 ± 4.4 *Px:* 76.4 ± 4.3	400 IU tocopherol every night for 6 months	20 mg pravastatin each night for 6 months	Global Health Perception Question, GDS, Assessment of Living Skills and Resources questionnaire< Wechsler Adult Intelligence Scale-R, Sleep Dysfunction Scale	12 months Data at 6 months used for meta-analysis.	No significant changes in health perception, depression, physical function, cognition or sleep dysfunction occurred After 6 months, GDS showed improvement from 2.00 ± 2.27 to 1.17 ± 1.20 in the tocopherol group versus deprovement from 1.20 ± 2.31 to 1.35 ± 2.37 in placebo (SMD: –0.64, 95%CI: –1.33, 0.04)†
Lohr et al. (1988) [35]	RCT	15	Mean age of 44 ± 18 (range 19–71) Participants have chronic schizophrenia (n = 9) or schizoaffective disorder (n = 6) and persistent tardive dyskinesia for at least 1 year Participants were kept on constant doses of neuroleptic and anticholinergic medications throughout the study	Alpha-tocopherol 400 IU 1st week: once in the morning 2nd week: twice a day 3rd and 4th week: thrice a day	Placebo	BPRS, a modified version of the Abnormal In- voluntary Movement Scale (AIMS) with a score range of 0 to 36, a modified version of the Simpson-Angus Scale for Extra- pyramidal Side Effects (SAS) with a score range of 0 to 24	10 weeks Data at 4 weeks used for meta-analysis.	Improvement in depression and anxiety with alpha-tocopherol versus placebo, both of which were not statistically significant After 4 weeks, BRPS depression subscale showed better results in the alpha-tocopherol group of 1.4 ± 2.4 versus 3.1 ± 3.2 in placebo (SMD: –0.58, 95%CI: –1.32, 0.15)† Anxiety subscale similarly showed better scores of 1.1 ± 1.9 in the alpha-tocopherol group versus 2.4 ± 2.5 in placebo (SMD: –0.57, 95%CI: –1.30, 0.16)‡
Mazloom et al. (2013) [36]	RCT	41	*Vitamin C:* 47 ± 8.93 *Vitamin E:* 48 ± 6.28 *Placebo:* 46.61 ± 7.58 Type 2 diabetic patients receiving standard oral hypoglycemic agents	Vitamin E capsule, 400 IU One capsule per day for 6 weeks	Vitamin C capsule, 1000 mg Placebo capsule (acetate cellulose), 1000 mg	DASS	6 weeks Data at 6 weeks used for meta-analysis.	No significant difference in depression or anxiety with Vitamin E versus placebo After 6 weeks, depression deproved from 21.92 ± 6.54 to 23.78 ± 6.11 in Vitamin E group versus 20.23 ± 5.65 to 21.15 ± 8.09 with placebo (SMD: 0.20, 95%CI: –0.55, 0.96)† Anxiety worsened from 31.07 ± 6.24 to 34.28 ± 7.54 with Vitamin E versus improvement from 28.69 ± 9.40 to 27.92 ± 8.73 with placebo (SMD: 0.75, 95%CI: –0.04, 1.53)‡
Malaguarnera et al. (2016) [37]	RCT	62	*Tx: 47.2* ± 3.7 yo Pb: 45.8 ± 3.9 yo 58% male Patients with chronic Hepatitis C, who are treated with Peg-IFN-alpha and RBV	94 mg silybin, 30 mg vitamin E, 194 mg phospholipids Three times a day for 12 months	Placebo	BDI, BPRS, Work Ability Index	12 months Data at 6 months used for meta-analysis.	Significant reduction in depression and anxiety were observed in the intervention group versus placebo group After 6 months, BDI deproved from 30.7 ± 7.1 to 34.6 ± 7.1 with vitamin E versus 30.8 ± 6.9 versus 46.7 ± 6.8 with placebo (SMD: –2.69, 95%CI: –3.38, –1.99)† STAI improved from 50.8 ± 7.9 to 50.4 ± 7.2 with Vitamin E but deproved from 50.1 ± 7.6 to 60.4 ± 7.7 with placebo (SMD: –2.19, 95%CI: –2.82, –1.55)‡
Lu et al. (2009) [38]	RCT	756	55–91 54.3% male patients with Amnestic Mild Cognitive Impairment (aMCI)	Donepezil, 10 mg Duration of intervention: 3 years	Vitamin E, 2000 IU Placebo	BDI and time to diagnosis of possible or probable AD according to NINCDS-ADRDA criteria	Every 6 months, up to 36 months Data at 6 months used for meta-analysis.	No significant improvement of depression with Vitamin E versus placebo After 6 months, BDI improved from 14.1 ± 4.3 to 11.3 ± 6.0 with Vitamin E versus 13.4 ± 3.8 to 11.4 ± 5.2 with placebo (SMD: –0.24, 95%CI: –0.58, 0.10)†
Adler et al. (1999) [39]	RCT	107	Patients with tardive dyskinesia	1600 IU per day of d-vitamin E	Placebo	BPRS	2 years Data at 12 months used for meta-analysis.	No significant effects on BPRS (SMD: 0.32, 95%CI: –0.06, 0.71) BPRS subscale scores for depression and axiety not reported No significant adverse events noted
Meyer et al. (2013) [40]	RCT	95	18–75 years Major Depression	Eight 1 g capsules yielding 250 mg DHA, 70 mg EPA, 10 mg vitamin E per day for 16 weeks	Placebo	HDRS, BDI	16 weeks	Trial did not show beneficial effects of DHA Quantitative results were not available for meta-analysis

Abbreviations: RCT, randomised-controlled trial; DHA, docosahexaenoic acid; EPA, eicosapentaenoic acid; IU, international units; BDI, Beck’s Depression Inventory; HDRS, Hamilton Depression Rating Scale; MADRS, Montgomery-Asberg Depression Rating Scale; BPRS, Brief Psychiatric Rating Scale; GDS, Geriatric Depression Scale; STAI, State-trait Anxiety Inventory; DASS, Depression Anxiety and Stress Scale; SMD, standardised mean difference; 95% CI, 95% confidence interval. * Mean (standard deviation) reported unless otherwise stated. † This outcome was used in meta-analysis of depression. ‡This outcome was used in meta-analysis of anxiety. Depression.

## Data Availability

No new data were created or analyzed in this study. Data sharing is not applicable to this article.
